# *NT5E* (CD73) is epigenetically regulated in malignant melanoma and associated with metastatic site specificity

**DOI:** 10.1038/bjc.2012.95

**Published:** 2012-03-27

**Authors:** H Wang, S Lee, C Lo Nigro, L Lattanzio, M Merlano, M Monteverde, R Matin, K Purdie, N Mladkova, D Bergamaschi, C Harwood, N Syed, P Szlosarek, E Briasoulis, A McHugh, A Thompson, A Evans, I Leigh, C Fleming, G J Inman, E Hatzimichael, C Proby, T Crook

**Affiliations:** 1 Wellcome Trust Centre for Molecular Medicine, Division of Medical Sciences, Clinical Research Centre and Department of Dermatology, Ninewells Hospital, University of Dundee, Dundee, DD1 9SY, UK; 2 Immunobiology Laboratory, London Research Institute, Lincoln's Inn Fields Laboratories, 44 Lincoln's Inn Fields, London WC2A 3LY, UK; 3 Laboratory of Cancer Genetics and Translational Oncology, Oncology Department, S Croce General Hospital, Cuneo, Italy; 4 Centre for Cutaneous Research, Blizard Institute, Barts and the London School of Medicine and Dentistry, Queen Mary University of London, 4 Newark Street, London E1 2AT, UK; 5 Faculty of Medicine, Imperial College London, Neuroscience Centre, Charing Cross Hospital, London, UK; 6 Department of Medical Oncology, Bart's and the London School of Medicine, Charterhouse Square, London, UK; 7 Department of Hematology and Interscience Molecular Oncology Laboratory, University Hospital of Ioannina Cancer Biobank Center, Street Niarchou Avenue, Ioannina, Greece; 8 Division of Cancer Research, Medical Research Institute, University of Dundee, Ninewells Hospital and Medical School, Dundee, DD1 9SY, UK

**Keywords:** melanoma, NT5E, epigenetics, metastasis

## Abstract

**Background:**

Novel prognostic biomarkers and therapeutic strategies are urgently required for malignant melanoma. Ecto-5-prime-nucleotidase (*NT5E*; CD73) overexpression has been reported in several human cancers. The mechanism(s) underlying deregulated expression and the clinical consequences of changes in expression are not known.

**Methods:**

We used RT–PCR, qPCR, methylation-specific PCR and pyrosequencing to analyse expression and regulation of *NT5E* in malignant melanoma cell lines and primary and metastatic melanomas.

**Results:**

*NT5E* is subject to epigenetic regulation in melanoma. *NT5E* mRNA is downregulated by methylation-dependent transcriptional silencing in the melanoma cell lines SKMel2, SKMel23, WM35, Mel501, Mel505 and C81–61 and expression is reactivated by azacytidine. In contrast, the CpG island is unmethylated and the gene expressed in cultured normal melanocytes. In clinical cases of melanoma, methylation in the *NT5E* CpG island occurs in both primary and metastatic melanomas and correlates with transcriptional downregulation of *NT5E* mRNA. Relapse with metastatic disease, particularly to the visceral sites and brain, is more common in primary melanomas lacking *NT5E* methylation. Primary melanomas with methylation in *NT5E* show limited metastatic potential or more commonly metastasise predominantly to nodal sites rather than viscera and brain (*P*=0.01).

**Conclusion:**

Deregulation of *NT5E* expression in melanoma occurs via epigenetic changes in the *NT5E* CpG island. Confirmation of our results in larger clinical series would support the candidacy of *NT5E* as a clinical biomarker in melanoma, which could be applied in both primary and relapsed disease. Inhibition of NT5E may have therapeutic potential in melanoma, particularly in patients with more aggressive disease metastatic to viscera or the brain.

Melanoma is an increasingly common, aggressive neoplastic disorder of melanocytes, with a remarkable propensity to disseminate and form metastases in distant organs sites, including the bone, lungs, liver and brain. To date, no adjuvant therapy after control of the primary tumour has shown clear benefit for patients with high-risk primary melanoma and the prognosis for patients with metastatic disease, particularly in visceral sites and brain is frequently very poor. Despite empirical testing of numerous chemotherapeutic regimens and targeted therapies, melanoma remains an essentially incurable disorder in patients with disseminated disease, with survival typically of a few months. Early detection and effective treatment of melanoma at early stages are urgently required, particularly as the incidence of the disease increases and the demography relentlessly moves towards a young patient group. The unique biology of the melanocyte and highly individual pathobiology of melanoma implies that a large number of genes may incur genetic or epigenetic change during the development of melanoma.

Epigenetics is the study of changes in gene expression without concomitant changes in gene structure and is now well recognised as an important mechanism by which the expression of genes may be modified in cancer (Lopez *et al*, 2009). Although the genome undergoes a process of global hypomethylation during tumorigenesis, specific CpG islands become hypermethylated with associated silencing of genes. Such methylation-dependent transcriptional silencing is a common finding in many human tumours and genes subject to this process are usually tumour suppressors. Methylation of the majority of genes is specific to neoplasia. Furthermore, methylated genomic DNA is stable and the technology for detection of methylated genomic DNA is sensitive and readily amenable to automation. Taken together, these are attractive properties for the use of methylated genomic DNA as a disease biomarker, provided markers of sufficient specificity for routine clinical use can be identified and their utility verified in large patient populations.

Ecto-5-prime-nucleotidase (*NT5E*; CD73) has a number of documented functions, the best characterised of which is catalysis of the conversion of extracellular purine 5′ mononucleotides to membrane-permeable nucleosides, the preferred substrate being AMP, resulting in local generation of adenosine. Evidence from a number of experimental systems suggests that expression of NT5E may be important in increasing the invasive and metastatic properties of some cancer cells and overexpression of NT5E may contribute to progression of cancer via generation of adenosine (Wang *et al*, 2011). Further, NT5E-dependent production of adenosine by cancer exosomes causes suppression of T-lymphocyte function (Clayton *et al*, 2011). Although these observations all support an oncogenic role for NT5E, other properties may be consistent with a potential tumour suppressor function. For example, knockdown of NT5E increases cell migration in specific cell types (Andrade *et al*, 2011). NT5E is overexpressed in some human tumours, but the mechanism(s) causing overexpression have not been defined. In the present study, we show that *NT5E* (CD73) is subject to methylation-dependent transcriptional silencing in melanoma, with potential clinical and therapeutic implications.

## Materials and Methods

### Cell lines

Melanoma cell lines were routinely grown in Dulbecco's modified Eagles's medium or Roswell Park Memorial Institute medium, supplemented with 10% fetal bovine serum. Primary, normal human melanocytes were purchased from Clonetics (Lonza Group, Basel, Switzerland) and grown according to the manufacturer's instructions. All cells were incubated at 37 °C and 5% CO_2_, with regular mycoplasma contamination test. The characteristics of the cell lines are summarised in Table 1.

### Demethylation

Demethylating treatment was as described previously (Lee *et al*, 2010). Briefly, cells were treated with 5-*μ*M azacytidine (AZA; Sigma, Gillingham, UK) for 7 days. Cells were split every 2–3 days with the addition of fresh drug. After drug treatment, cells were harvested for qPCR.

### Tissues

The study received approval from the Local Research Ethical Committee, East London and City Health Authority and the Tayside Tissue Bank under delegated authority from the Tayside Local Research Ethics Committee. Tissues were obtained under written, informed consent. Melanomas and control benign nevi were from the Pathology archives of Bart's and the London NHS Trust and from the Tayside Skin Cancer Tissue Bank. All tissues underwent histopathological review before inclusion in the study to (i) confirm the diagnosis of melanoma; (ii) to confirm Breslow thickness (BT); and (iii) to verify sufficient representation of neoplastic cells. Melanomas were both primary, cutaneous melanomas and metastatic lesions. Surgical excision was performed according to routine protocols of clinical care. BT and melanoma sub-type is shown in Figure 4 for the primary melanomas. Recurrent melanomas were typically from lymph node resections, (where performed) sentinel lymph node biopsies or cutaneous recurrences. Benign nevi were from surgical excisions and confirmed by histopathological analysis to contain no neoplastic cells.

### Analysis of methylation

Genomic DNA was obtained from formalin-fixed paraffin-embedded tissue sections by extended digestion in Proteinase K followed by phenol extraction. Genomic DNA from cell lines and tissue samples was subjected to bisulphite modification using the EZ DNA Methylation Kit (Zymo, Irvine, CA, USA) according to the manufacture's instructions. Methylation in the CpG island of the *NT5E* gene was analysed with pyrosequencing technology, in which the degree of methylation at each CpG position in a sequence is determined from the ratio of T and C, following bisulphite modification. Primer sequences were as follows: forward 5′-GTATTAGGGTATTATTTGGTTTAT-3′ reverse 5′-BIOT-CTTACCACACTCTACCATCC-3′ fragment size: 170 bp.

Polymerase chain reaction conditions were as follows: 95 °C for 10 min, 95 °C for 30 s/54 °C for 30 s/72 °C for 40 s for 40 cycles, 72 °C for 7 min. Polymerase chain reaction products were resolved through 2% agarose gels, visualised using a transilluminator, then analysed by pyrosequencing using the Biotage Sample Prep kit and using the forward primer for sequencing. Analysis of percentage methylation at each CpG dinucleotide was performed using CpG Software (Qiagen, Crawley, UK). Placental DNA was used as negative control of methylation (0% average methylation) and a commercial methylated DNA (Millipore, Watford, UK) was used as positive control (98% average methylation). Methylation was also analysed by methylation-specific PCR (MSP). DNA (0.5 *μ*g) was modified by sodium bisulphite using the Zymo EZ DNA Methylation kit (Zymo). Bisulfite-modified DNA was used as a template for PCR with primers specific for methylated or unmethylated alleles. CpGenome Universal Methylated DNA (Chemicon Europe, Chandlers Ford, Hampshire, UK) and normal human unmethylated DNA were used as positive and negative controls, respectively, in each experiment. MSP primer sequences: M forward primer: 5′-TATTTTATGAACGTTTTGCGTTACG-3′ M reverse primer: 5′-CTAAACTTACCACACTCTACCATCCG-3′ U forward primer: 5′-ATTTTATGAATGTTTTGTGTTATGA-3′ U reverse primer: 5′-AACTTACCACACTCTACCATCCACT-3′.

### Gene expression

For qPCR analysis of expression, total RNA was isolated musing the Recover All Total Nucleic Acid Isolation (Ambion, Life Technologies Italia, Monza, Italy). In all, 25 *μ*l PCR reactions were performed using 50 ng of cDNA obtained by reverse transcription. Amplification and analysis were done according to the manufacturer's protocol in 96-well plates in an ABI PRISM 7000 Sequence Detection System (Applied Biosystems, Life Technologies Italia, Monza, Italy) and the pre-cast ‘TaqMan Gene Expression Assays’ (Applera, https://products.appliedbiosystems.com/) for NT5E (Hs001573922_m1). Quantification of the target transcript was performed in comparison to the reference transcript *β*2microglobulin (Hs99999907_m1), using the ‘delta-delta Ct method for comparing relative expression results in real-time PCR as outlined by PE Applied Biosystems (Perkin Elmer, Forster City, CA, USA).

### Statistics

*NT5E* CpG island methylation status and presence or types of metastasis were assessed for associations with the Fisher's exact test. All of the statistical analyses were performed using Prism 5 (GraphPad software, Inc., La Jolla, CA, USA).

## Results

### *NT5E* expression is deregulated in highly metastatic C8161 melanoma cells by loss of CpG island methylation from the parental C81–61 cells

**Figure 1:**
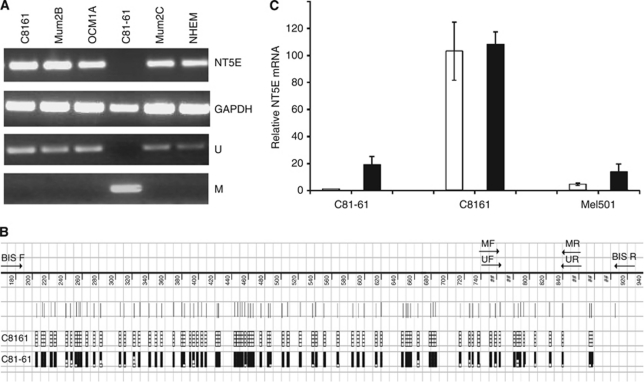
*NT5E* expression is silenced by methylation in C81–61 cells, but deregulated in highly metastatic isogenic C8161 cells. (**A**) Expression of *NT5E* correlates inversely with CpG methylation in C81–61 and C8161. The upper panels are RT–PCR analysis of *NT5E* and the control gene *GAPDH* in five melanoma cell lines and normal human melanocytes (NHEM) as indicated. *NT5E* mRNA is expressed abundantly in all cell lines except C81–61. Lower panels are MSP analysis of the *NT5E* CpG island in the same cell lines. There is complete methylation in C81–61 but no detectable methylation in C8161. (**B**) Bisulphite sequencing analysis of the *NT5E* CpG island in C81–61 and C8161 cell lines. Bisulphite sequencing was performed as described in Materials and Methods and the figure shows a diagrammatic representation of the *NT5E* CpG island. CpG sites are shown as vertical lines. Methylated CpG dinucleotides are shown as black blocks, unmethylated CpGs as open blocks. Five levels of methylation are indicated: 0-no black blocks; 1–25%-1 black block; 25–50%-2 black blocks; 50–75%-3 black blocks; 75–100%-4 black blocks. Positions of the MSP and bisulphite-sequencing primers are indicated. The CpG island is almost completely methylated in C81–61 cells and entirely unmethylated in C8161, consistent with expression of *NT5E* mRNA in each cell line. (**C**) Expression of *NT5E* mRNA is reactivated by demethylation. Exponentially growing C81–61, C8161 and Mel501 cells were treated with 5′azacytidine (5′ AZA) (black blocks) or untreated (open blocks). cDNA was prepared and expression of *NT5E* mRNA determined as described in Materials and Methods.

We analysed expression of *NT5E* mRNA in the paired malignant melanoma cell lines C81–61 (low metastatic potential) and the highly metastatic isogenic derivative C8161 (high metastatic potential). We also analysed expression in three additional melanoma cell lines and in normal human melanocytes. *NT5E* mRNA was abundantly expressed in normal melanoyctes, in C8161 cells and in each of the three other melanoma cell lines. However, expression was not detectable by RT–PCR in C81–61 cells (Figure 1A). A CpG island is located at the 5′ end of the *NT5E* gene (Figure 1B). To test whether aberrant methylation might be the basis for the low expression of NT5E mRNA in C81–61, we performed bisulphite sequence analysis of the CpG island in C81–61 (absent expression) and the C8161 derivative (high expression). The CpG island was entirely unmethylated in C8161 but densely methylated in C81–61, consistent with aberrant methylation underlying the absence of expression (Figure 1B). On the basis of these observations, we designed primers for MSP and analysed methylation in the cell lines. Methylation was only observed in C81–61 consistent with bisulphite sequencing and absence of expression in this cell line (Figure 1A). To further verify that methylation is the mechanistic basis for *NT5E* downregulation, we treated C81–61 and C8161 cells with the demethylating agent AZA and measured mRNA levels. *NT5E* mRNA was increased by AZA in C81–61 but not in C8161 (Figure 1C).

### *NT5E* is frequently methylated in melanoma cell lines

**Figure 2:**
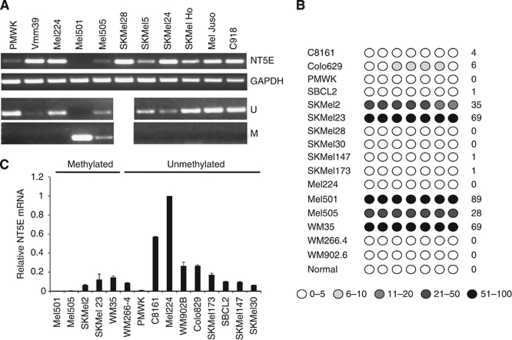
Analysis of *NT5E* expression and methylation in malignant melanoma. (**A**) Expression of *NT5E* correlates inversely with CpG methylation in melanoma cell lines. The upper panels are RT–PCR analysis of *NT5E* and the control gene *GAPDH* in melanoma cell lines as indicated. Lower panels are MSP analysis of the *NT5E* CpG island in the same cell lines. There is complete methylation in Mel501 and partial methylation in Mel505. (**B**) Pyrosequencing analysis of the *NT5E* CpG island in a panel of melanoma cell lines. Pyrosequencing was done as described in Materials and Methods. The level of methylation is represented by the intensity of shading in the circles, each of which represents an individual CpG dinucleotide in the amplified fragment. The mean % CpG methylation in the amplified fragment is also shown. (**C**) qPCR analysis of *NT5E* mRNA levels in panel of melanoma cell lines. Data shown are means of at least three duplicates±1 s.d.

**Figure 3:**
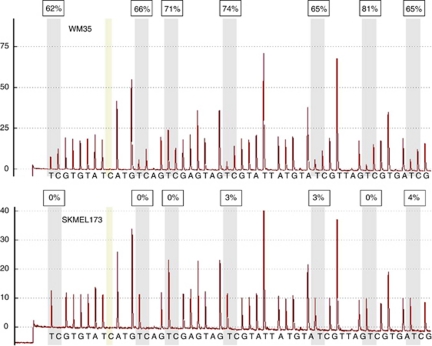
Pyrosequencing analysis of the *NT5E* CpG island in WM35 and SKMel173 cell lines. The figure shows representative pyrograms. The shaded C residue is the control for bisulphite conversion: there must be no peak at this residue (non CpG cytosine/guanine), confirming 0% cytosine incorporation. % Methylation at each CpG is indicated.

We next analysed *NT5E* expression in a panel of additional melanoma cell lines. By RT–PCR, *NT5E* mRNA was expressed in the majority of the cell lines analysed but was undetectable in Mel501 and present only at low levels in Mel505 and PMWK (Figure 2A). To validate these observations, we performed analysis of *NT5E* expression and methylation in an independent panel of melanoma cell lines (which contained a subset of the lines analysed by semi-quantitative methods) using qPCR and pyrosequencing. Representative pyrograms are shown in Figure 3. In total, there was methylation in 6/16 cell lines (C81–61 (bisulphite sequencing), SKMel2, SKMel23, WM-35, Mel501 and Mel505; Table 1; Figure 2B). There was a good correlation between the presence of methylation in the *NT5E* CpG island and absent or reduced expression of the mRNA (Figure 2C and D). As in C81–61 cells, demethylation by exposure to AZA caused an increase in *NT5E* mRNA levels in Mel501 cells (Figure 1C).

### The *NT5E* CpG island is methylated in clinical cases of melanoma

These results show that expression of *NT5E* is regulated epigenetically in melanoma cell lines and prompted us to test whether the *NT5E* CpG island is methylated *in vivo*. We first analysed, using MSP, CpG methylation in a series of 52 unselected, histologically confirmed melanomas. Consistent with studies in cell lines, there was methylation in the *NT5E* CpG island in 22/52 (42%) biopsies, as detected by MSP. We validated the frequency of methylation by performing pyrosequencing on a subset of the cases analysed by MSP and this showed a concordance in 8/9 melanomas.

### Methylation of *NT5E* is associated with specific sites of metastasis

**Figure 4:**
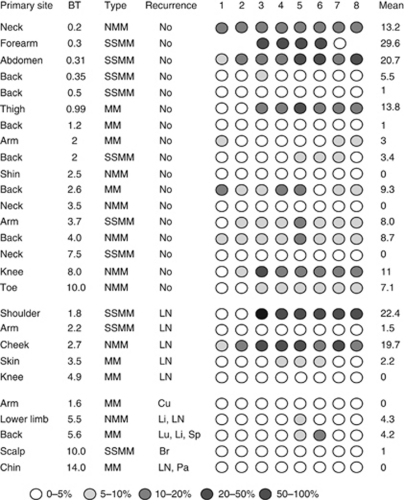
Methylation in the *NT5E* CpG island in primary malignant melanoma. Pyrosequencing analysis of the *NT5E* CpG island in primary malignant melanomas from sun-exposed sites was done as described in Materials and Methods. The level of methylation is represented by the intensity of shading in the circles, each of which represents an individual CpG dinucleotide in the amplified fragment. The mean % CpG methylation in the amplified fragment is also shown. The anatomical site of the primary lesion is indicated, together with the Breslow thickness (BT) in mm and the melanoma type (where available): NMM=nodular malignant melanoma; MM=malignant melanoma, sub-type not available; SSMM=superficial spreading malignant melanoma. Site(s) of metastasis (if applicable) are indicated: Br=brain; Cu=cutaneous; Li=liver; LN=lymph Node; Lu=lung; Or=orbit; Pa=parotid gland; Sp=spleen.

We further investigated methylation in clinical cases of melanoma by analysis of a well-characterised series of primary and metastatic melanomas using pyrosequencing. As controls, we first tested a series of benign nevi. This revealed a mean % methylation of 8.3% in the nevi. Accordingly, we designated a value of 9% as the cut off for methylation in melanomas and proceeded to analyse *NT5E* CpG methylation in 27 primary melanomas from various sun-exposed sites (Figure 4). Methylation was detected in 8/27 cases (29%), a frequency comparable to the initial series of cases analysed by MSP. Methylation was more common in nonrelapsing cases, although this did not reach statistical significance because of the small number of cases (6/17 *vs* 2/10; *P*=0.23). In the 10 cases with subsequent metastatic relapse, five were in visceral sites (the brain, liver, lung and parotid) and five in nonvisceral sites (Figure 4). The *NT5E* CpG island was methylated in 0/5 cases with visceral metastasis, whereas 2/5 cases with nonvisceral metastasis were methylated in the *NT5E* CpG island (*P*=0.16). We noted that the two relapsing cases with methylation were both lymph node metastases.

### NT5E methylation protects against metastasis in high-risk primary melanomas

The 27 initially analysed cases included 17 with a BT >2 mm, of which 8/17 had relapsed at the time of censor. Relapse was more common in cases lacking *NT5E* methylation (1/8 methylated *vs* 7/8 unmethylated), although again because of the small number of cases this difference did not reach statistical significance (*P*=0.13).

### *NT5E* methylation in nodal/cutaneous metastases predicts risk of visceral metastases

**Figure 5:**
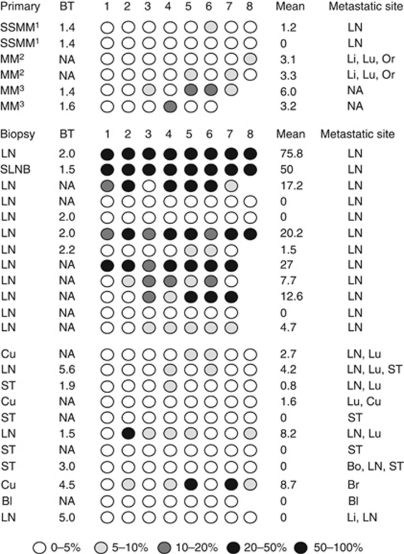
*NT5E* CpG methylation in cutaneous lesions or LN predicts visceral *vs* nonvisceral sites of distant metastasis. Pyrosequencing was performed as described in Materials and Methods. The level of methylation is represented by the intensity of shading in the circles, each of which represents an individual CpG dinucleotide in the amplified fragment. The mean % methylation is also shown. The site of biopsy is indicated, together with the site of metastasis: Bl, Bo, Br, Cu, Li, LN, Lu, Or, Pa and ST. Also shown (where available) is the Breslow thickness (BT) of the primary melanoma from which the metastatic lesions derived. The superscripts^1,2,3^ denote paired cutaneous biopsies from the same patients. Note that in each case with paired biopsies, the level of methylation is similar in the two biopsies. Abbreviations: Bl=bladder; Bo=bone; Br=brain; Cu=cutaneous; Li=liver; LN=lymph node; Lu=lung; NA=not available; Or=orbit; ST=soft tissue.

These results prompted us to further examine the apparent inverse correlation between methylation in *NT5E* and visceral metastasis. We performed pyrosequencing in 26 metastatic lesions from LN or cutaneous sites confirmed by histopathology to be malignant melanoma, which arose in patients with a previous primary melanoma (Figure 5). The *NT5E* CpG island was methylated in 6/26 cases, using the methylation cutoff of 9% previously established in benign nevi. Metastasis to visceral sites, including bladder, brain, lung and liver, occurred in 12 patients and in all 12 the *NT5E* CpG island in the sampled lymph node/cutaneous metastasis was unmethylated (*P*=0.01, *OR*=19; Figure 5).

## Discussion

Despite advances in understanding of the fundamental biology of the melanocyte and development of therapeutic agents, including the BRAF inhibitor Vemurafenib and the monoclonal antibody Ipilimumab, metastatic malignant melanoma remains an incurable disease with a poor prognosis. As such, additional mechanistic insights to growth and progression of melanoma and novel therapeutic strategies are clearly required. Prognosis at diagnosis of patients with primary melanomas is estimated largely by histopathological parameters, such as BT, presence of ulceration and lympho-vascular invasion, but the ability of these to accurately predict clinical outcomes is limited. As such, molecular genetic biomarkers would be valuable in assessing risk of metastatic relapse to viscera and/or brain: events that are associated with poor clinical outcomes. Similarly, initial relapse in cutaneous or nodal sites is common. Such relapses are typically managed by surgical excision. This is not only therapeutic but provides, as part of routine clinical management a source of tissue for assessment of prognosis, assuming robust biomarkers to predict the risk of later metastatic relapse to viscera and/or brain can be identified. Such biomarkers would be immensely useful to inform clinical management of such patients. Here, we report that expression of *NT5E* (CD73) is regulated epigenetically in malignant melanoma. We show that expression is downregulated by aberrant methylation in the CpG island located in the 5′ regulatory region of the *NT5E* gene in melanoma cell lines and clinical melanomas, and we present preliminary evidence that methylation in the *NT5E* CpG island may have utility as a biomarker of risk of visceral/brain metastasis, both in primary melanoma and in cutaneous and/or nodal recurrence. To the best of our knowledge, this is the first report of epigenetic regulation of this gene in human neoplasia, although a single study has identified upregulation of *NT5E* by chemical demethylation in a gastric carcinoma cell line, consistent with methylation-dependent transcriptional silencing (Yamashita *et al*, 2006).

In the initial studies, we showed that *NT5E* mRNA was abundantly overexpressed in C8161 cells, which have a highly invasive and metastatic phenotype, but was not expressed in (isogenic) parental C81–61 cells, implying that deregulation of *NT5E* contributes to the greater metastatic potential of C8161 cells. Bisulphite sequencing and MSP revealed that the *NT5E* CpG island was fully methylated in parental C81–61 cells but was unmethylated in C8161. Taken together with reactivation of expression by AZA, these findings imply that loss of methylation is the mechanistic basis for deregulation of *NT5E* in C8161 cells. These results not only support a role for NT5E in the more metastatic phenotype of C8161 cells but are also consistent, in a wider context, with mechanistic models in which epigenetic changes in key genes contribute to malignant progression. More commonly in neoplasia, methylation increases in the CpG islands of genes as tumour suppressors are silenced and cancer cells acquire progressively more malignant properties (Lopez *et al*, 2009). *NT5E*, however, appears to be a gene in which absence of methylation contributes to a more malignant phenotype. Previous studies on the expression of NT5E have reported overexpression of the gene in invasive melanoma cell lines (Sadej *et al*, 2006a, 2006b). It was, therefore, unexpected to find that the gene was silenced by methylation in some melanoma cell lines. The selective pressure favouring downregulation of *NT5E* in some cell lines is not clear. However, it has been previously shown that *NT5E* mRNA is upregulated during differentiation of melanoma cell lines (Kim *et al*, 2006). Further, knock-down of *NT5E* causes increased cell migration in some cell types (Andrade *et al*, 2011). It is therefore possible that, perhaps in early disease, methylation-dependent silencing of *NT5E* might block differentiation and/or promote migration in a subset of melanomas.

The studies of clinical melanomas revealed a frequency of *NT5E* CpG methylation similar to cell lines, using MSP and pyrosequencing. We show that methylation in both primary melanomas and in cutaneous and lymph node metastases influences the clinical behaviour of the disease, cases with methylation being less likely to metastasise to visceral sites such as the lung and liver and brain, whereas cases with methylation more commonly metastasise to nodal sites. Of particular interest, in patients with primary melanomas of BT >2 mm, which are at high increased risk of metastasis, methylation in the *NT5E* CpG island was associated with a lower risk of metastasis to visceral sites and brain than cases lacking *NT5E* methylation. The potential biomarker utility of *NT5E* methylation was also evident in relapsed/metastatic lesions from cutaneous or nodal sites, cases with *NT5E* methylation being more likely to subsequently metastasise to viscera or brain if the *NT5E* CpG island was unmethylated. Such nonvisceral metastatic lesions are typically treated as part of routine clinical management by surgical resection and tissue obtained from these procedures would clearly be amenable to analysis of biomarkers such as NT5E. As normal stromal skin tissue also expresses high levels of NT5E (http://www.proteinatlas.org/ENSG00000135318), immunohistochemical analysis of NT5E may not be prognostically informative. Importantly, we have shown in the present study that *NT5E* methylation levels are low in normal melanocytes and benign nevi, emphasising the specificity of *NT5E* methylation for neoplastic melanocytes. In some cases, surgically excised melanomas contain a significant normal tissue component, potentially limiting the biomarker utility of detection of *NT5E* CpG methylation, but this could be readily overcome by micro-dissection of tissue sections before methylation analysis. The sensitivity of methylation analysis and its specificity for neoplasia implies that *NT5E* methylation may be more informative than immunohistochemistry as a prognostic biomarker. Studies to address this question are in progress.

Our results are consistent with animal models in which NT5E overexpression is associated with aggressive behaviour of cancers (Stagg *et al*, 2011) and in mouse models in which the growth of primary tumours and formation of metastases are reduced in mice lacking NT5E (Yegutkin *et al*, 2011). Aside from the potential biomarker utility of *NT5E*, the likely role of NT5E in driving metastatic disease in a proportion of cases implies that NT5E may be a viable therapeutic target in melanoma, particularly in clinically challenging cases with visceral and/or brain metastases. Evidence from animal models suggests that inhibition of both host and tumour cell NT5E has anti-cancer effects (Wang *et al*, 2011; Yegutkin *et al*, 2011), emphasising the likely value of inhibition of NT5E. Our results suggest that use of NT5E inhibitors should be informed, at least in part, by analysis of the methylation status of the CpG island.

In conclusion, we show that methylation in the *NT5E* CpG island is an important determinant of metastatic potential of malignant melanoma. Confirmation of these results in larger, independent series would support the candidacy of *NT5E* as a potentially clinically useful epigenetic biomarker to inform management of melanoma patients, particularly those with high-risk primary lesions and in those with nodal and/or cutaneous recurrences/metastases.

## Figures and Tables

**Table 1:** Characteristics of melanoma cell lines

**Cell line**	**Description**	**B-raf/N-ras**	**NT5E**
HEMA	Normal human melanocytes	Wt/Wt	U
SBCL2	RGP	Wt/Q61L	U
PMWK	Early RGP	Wt/Wt	U
WM-35	RGP	V600E/Wt	M
WM-902B	SSM VGP	V600E/Wt	U
Mel224	VGP	Wt/Q61R	U
Mel505	VGP	Wt/Wt	M
WM-266-4	Metastatic melanoma	V600D/Wt	U
SKMel2	Metastatic melanoma	Wt/Q61R	M
SKMel23	Metastatic melanoma	G466A/Wt	M
SKMel28	Metastatic melanoma	V600E/Wt	U
SKMel30	Metastatic melanoma	Wt/Q61R	U
SKMel147	Metastatic melanoma	Wt/Q61R	U
SKMel173	Metastatic melanoma	Wt/Q61K	U
Mel501	Metastatic melanoma	V600E/Wt	M
COLO-829	Metastatic melanoma	V600E/Wt	U
C81-61	Metastatic melanoma	Wt/Wt	M
C8161	Metastatic melanoma	Wt/Wt	U

Abbreviations: M=methylated; RGP=radial growth phase; SSM=superficial spreading melanoma; U=unmethylated; VGP=vertical growth phase melanoma; Wt=wild-type.
